# Severity of SARS-CoV-2 alpha variant (B.1.1.7) in England

**DOI:** 10.1093/cid/ciab754

**Published:** 2021-09-06

**Authors:** Daniel J Grint, Kevin Wing, Catherine Houlihan, Hamish P Gibbs, Stephen J W Evans, Elizabeth Williamson, Helen I McDonald, Krishnan Bhaskaran, David Evans, Alex J Walker, George Hickman, Emily Nightingale, Anna Schultze, Christopher T Rentsch, Chris Bates, Jonathan Cockburn, Helen J Curtis, Caroline E Morton, Sebastian Bacon, Simon Davy, Angel Y S Wong, Amir Mehrkar, Laurie Tomlinson, Ian J Douglas, Rohini Mathur, Brian MacKenna, Peter Ingelsby, Richard Croker, John Parry, Frank Hester, Sam Harper, Nicholas J DeVito, Will Hulme, John Tazare, Liam Smeeth, Ben Goldacre, Rosalind M Eggo

**Affiliations:** 1 Faculty of Epidemiology and Population Health, London School of Hygiene & Tropical Medicine, UK; 2 Division of Infection and Immunity, University College London, London, UK; 3 The DataLab, Nuffield Department of Primary Care Health Sciences, University of Oxford, UK; 4 Faculty of Public Health and Policy, London School of Hygiene & Tropical Medicine, UK; 5 TPP, TPP House, 129 Low Lane, Horsforth, Leeds, UK

## Abstract

**Background:**

The SARS-CoV-2 alpha variant (B.1.1.7) is associated with higher transmissibility than wild type virus, becoming the dominant variant in England by January 2021. We aimed to describe the severity of the alpha variant in terms of the pathway of disease from testing positive to hospital admission and death.

**Methods:**

With the approval of NHS England, we linked individual-level data from primary care with SARS-CoV-2 community testing, hospital admission, and ONS all-cause death data. We used testing data with S-gene target failure as a proxy for distinguishing alpha and wild-type cases, and stratified Cox proportional hazards regression to compare the relative severity of alpha cases compared to wild type diagnosed from 16th November 2020 to 11th January 2021.

**Results:**

Using data from 185,234 people who tested positive for SARS-CoV-2 in the community (alpha=93,153; wild-type=92,081), in fully adjusted analysis accounting for individual-level demographics and comorbidities as well as regional variation in infection incidence, we found alpha associated with 73% higher hazards of all-cause death (aHR: 1.73 (95% CI 1.41 - 2.13; P<.0001)) and 62% higher hazards of hospital admission (aHR: 1.62 ((95% CI 1.48 - 1.78; P<.0001), compared to wild-type virus. Among patients already admitted to ICU, the association between alpha and increased all-cause mortality was smaller and the confidence interval included the null (aHR: 1.20 (95% CI 0.74 - 1.95; P=0.45)).

**Conclusions:**

The SARS-CoV-2 alpha variant is associated with an increased risk of both hospitalisation and mortality than wild-type virus.

## Introduction

The SARS-CoV-2 (COVID-19) variant of concern B.1.1.7, now called the alpha variant, was first identified in Kent, UK in autumn 2020.[1] Early analysis estimated that alpha is more transmissible than the original lineage and it became the dominant strain throughout the UK in early 2021.[2] Only a small number of alpha cases were originally identified by whole-genome sequencing. Certain PCR assays for SARS-CoV-2 that are used in three major laboratories in England do not amplify one of the spike protein gene targets in the alpha variant. Spike gene target failure (SGTF) was therefore adopted as a proxy for identifying alpha, and has been shown to have more than 95% sensitivity for alpha viruses during the period 16th November 2020 – 11th January 2021.[3]

While a number of studies have shown that alpha is associated with an overall higher case fatality than the original lineage,[4-8] studies specifically restricted to hospitalised patients have shown no difference in case fatality.[8,9] However, these findings are not necessarily contradictory as alpha may cause more severe disease leading to more people needing hospital admission, but may not be any more likely than the original lineage to cause death in those who already have severe disease requiring hospital care.

This study aims to bring these elements together in a consolidated analysis, following the pathway of disease from infection to hospital admission and death, in order to fully illuminate the association of the alpha variant with altered healthcare need and mortality.

## Methods

### Data platform

With the approval of NHS England, data were linked, stored and analysed securely within the OpenSAFELY electronic health records research platform.[10] OpenSAFELY holds electronic health records (EHRs) for 58 million individual registrations with a general practitioner (GP) in England, and in this study we use a subset of these who are registered at practices using the TPP EHR management system, which includes 24 million people, covering 40% of England's population. Primary care data include individual-level coded diagnoses, medications, vaccinations, and physiological parameters. These data were linked to key datasets to obtain: 1) SARS-CoV-2 community testing data through the Second Generation Surveillance System; 2) hospital admission data; 3) COVID-19 related intensive care unit (ICU) admission data; 4) all-cause registered deaths from the Office for National Statistics (ONS). More information on the OpenSAFELY analytical platform and data sources is available in supplement sections 1-3 and supplementary Table S1.

### Study design and population

We defined our study population as all who tested positive for SARS-CoV-2 in the community with data available on SGTF status, between 16 November 2020 and 11 January 2021. During this time alpha cases increased from a small minority as a proportion of all diagnosed SARS-CoV-2 infections in the UK, to the dominant majority. This period of cross-over from the original lineage to alpha presents the ideal cohort for comparison of the relative severity of alpha compared to wild type virus. The study period predates the emergence of the delta variant.

The primary exposure of interest was SGTF status. SGTF was taken as a proxy for identifying the SARS-CoV-2 alpha variant, and compared to cases without S-gene drop out (wild type).

### Statistical methods

The primary analysis used a Cox proportional hazards regression model stratified by geographic region, defined as upper tier local authority area (UTLA).[11,12] Stratification by region allowed a separate hazard function to be estimated for each region, with parameter estimates estimated over the full population. This degree of regional flexibility was included *a priori* to account for potentially non-proportional changes in pandemic incidence over time by region.

For analysis of all-cause mortality, follow-up began at the date of testing positive in the community for SARS-CoV-2 and was censored at 21 April 2021 or 7 days prior to receipt of a vaccination against SARS-CoV-2. Since illness which may lead to death would exclude the booking and administration of a vaccine, the 7 days prior to vaccination were censored to discount a potential immortal time bias.

For analysis of hospital admission, follow-up began at the date of testing positive for SARS-CoV-2 and was censored at 21 April 2021, the date of deregistration from GP practice, or 7 days prior to receipt of a vaccination against SARS-CoV-2.

For analysis of all-cause mortality among those admitted to hospital, follow-up began at the date of hospital admission and was censored at 21 April 2021 or 7 days prior to receipt of a vaccination against SARS-CoV-2. In England, the NHS vaccination programme for SARS-CoV-2 began in December 2020, consequently censoring on vaccination was rare in this study population. A further analysis of those admitted to hospital was performed on the population who spent time in the intensive care unit (ICU) during their hospital stay. This subset further conditions the population admitted to hospital to be those with severe illness who received intensive care.

Covariate adjustment was informed by consideration of causal pathways using a causal diagram. Subgroup analysis of the primary exposure was pre-specified for epidemiological week of SARS-CoV-2 diagnosis, comorbidity status, ethnicity in five categories, deprivation quintile, and age group.

Comorbidities were defined as in our prior work,[6] as the presence of codes in the patient's EHR indicating diagnoses. All codes and conditions are given in supplement section 4 Table S2.

A number of pre-specified sensitivity analyses were also performed including censoring all follow-up 28-days after SARS-CoV-2 diagnosis, restricting to the population with a minimum of 40 days' follow-up, and imputing missing data on ethnicity. Further information on analysis methods and full details of all pre-specified sensitivity and subgroup analyses is available in the study protocol (https://github.com/opensafely/SGTF-CFR-research/tree/master/docs/).

Absolute risk estimates were calculated from the marginal means of fully adjusted logistic regression models with the outcomes death by 28-days after a positive SARS-CoV-2 test, and hospital admission by 28-days after a positive SARS-CoV-2 test. In each case, the population was restricted to those who had a minimum of 28-days follow-up from the date of their positive SARS-CoV-2 test to the follow-up censor. In these models, deaths and hospital admissions beyond 28-days were censored. Vaccination prior to SARS-CoV-2 infection was an exclusion criterion in this analysis.

## Results

### Population characteristics

Our study population consists of 185,234 people testing positive for SARS-CoV-2 in the community between 16th November 2020 and 11th January 2021 for whom SGTF status was known (alpha=93,153; wild type=92,081). In the week beginning 16th November 2020 wild type cases accounted for 20,926/22,062 (94.9%) of total cases, by the week beginning 4th January 29,349/36,821 (79.7%) were alpha. Consequently, median follow-up time was shorter for alpha cases (102.0 days (interquartile range (IQR): 62.0-113.0) vs. 109.0 (71.0-136.0)), compared to wild type cases. Overall, alpha cases were concentrated in the East (37.5%), London (12.3%), and North West (11.0%), reflecting areas where the alpha epidemic began. Wild type cases were mainly from Yorkshire and the Humber (25.3%), the East Midlands (20.3%), and North West (16.0%) (Figure 1; supplementary Table S3).

People with the alpha variant were marginally younger overall (median age 37.0 vs. 38.0), with a smaller proportion of people aged 70-<80 (2.9% vs. 3.4%) and 80+ (0.9% vs. 1.7%), compared to wild type cases. Fewer people infected with the alpha variant had underlying comorbidities (1 comorbidity (10.4% vs. 11.6%); 2+ comorbidities (2.9% vs. 3.8%)), compared to those infected with wild type virus. The proportion of people identified as living in care homes was lower for alpha cases (0.1% vs. 0.4%). A lower proportion of people with the alpha variant lived in areas of the most deprived SES quintile (16.7% vs. 26.3%), whereas a higher proportion lived in areas of the least deprived SES quintile (22.1% vs. 17.4%), compared to people with wild type virus (Figure 1; supplementary Table S3).

### Case fatality

985 deaths of any cause were registered by 21st April 2021 (alpha: 500 (0.5%); wild type: 485 (0.5%)). In fully adjusted analysis, accounting for demographic factors, regional variation, and individual-level comorbidities, alpha was associated with 73% increased hazards of death (adjusted hazard ratio (aHR): 1.73 (95% confidence interval (CI): 1.41 - 2.13); *P*<0.0001) when compared to wild type (Figure 2). The increased hazard of death for alpha was consistent across all predefined subgroups and sensitivity analyses (supplementary Figure S1).

The absolute risk of death by 28-days post positive SARS-CoV-2 test for people with alpha was low for males (0.03% (95% CI: 0.01 - 0.04)) and females (0.01% (0.00 - 0.02)) aged 40 and below in the absence of comorbidities. However, the risk of death by 28-days was considerable for males (10.4% (7.1 - 13.7)) and females (6.0% (4.0 - 8.0)) aged 85 and over with alpha. In the presence of 2 or more comorbidities the risk of death by 28-days for those with alpha was increased for males (0.08% (95% CI: 0.02 - 0.13)) and females (0.04% (0.01 - 0.07)) aged 40 and below, and was particularly high for males (25.0% (19.5 - 30.4)) and females (15.7% (11.9 - 19.5)) aged 85 and above (Table 1).

### Admission to hospital

316 of 985 (32.1%) deaths registered in the study occurred without admission to hospital (alpha: 131; wild type: 185). People who died without hospital admission were older (median age 80.0 (IQR: 67.5 - 90.0) vs. 72.0 (62.0 - 81.0)), a higher proportion were female (51.9% vs. 37.7%), and a higher proportion were resident in a care home (31.6% vs. 6.0%), compared to deaths following admission to hospital (Figure 3).

4,910 people were admitted to hospital following a positive test for SARS-CoV-2 in our dataset (alpha: 2,721 (2.9%); wild type: 2,189 (2.4%)). Compared to the full study population, those admitted to hospital were older (median age 58.0 vs. 38.0), with more comorbidities (1 comorbidity: 27.4% vs. 11.0%; 2+ comorbidities: 21.9% vs. 3.4%). Among people admitted to hospital, those with alpha were younger (median age 57.0 (IQR: 47.0 - 68.0) vs. 59.0 (48.0 - 72.0)), and had fewer comorbidities (2+ comorbidities 19.3% vs. 25.2%), compared to those with wild type (supplementary Table S4).

In fully adjusted analysis, accounting for demographic factors, regional variation, and individual level comorbidities, alpha was associated with 62% increased hazards of hospital admission (aHR: 1.62 (95% CI: 1.48 - 1.78); $P$<0.0001) when compared to wild type (Figure 2). The increased hazard of hospital admission for alpha was consistent across all predefined subgroups and sensitivity analyses (supplementary Figure S2).

The absolute risk of hospital admission by 28-days post positive SARS-CoV-2 test for those with alpha was 1.1% for males (1.1% (95% CI: 1.01 - 1.24)) and 0.7% for females (0.74% (0.67 - 0.82)) aged 40 and below in the absence of comorbidities. However, the risk of hospitalisation was considerable for males (18.1% (14.9 - 21.2)) and females (12.8% (10.4 - 15.1)) aged 85 and above. In the presence of 2 or more comorbidities the risk of hospital admission for those with alpha was increased for males (3.3% (95% CI: 2.8 - 3.8)) and females (2.2% (1.9 - 2.5)) aged 40 and below, and high for males (38.8% (34.2 - 43.4)) and females (29.7% (25.7 - 33.8)) aged 85 and above (Table 2).

### Case fatality given hospital admission

There were 669 deaths among people admitted to hospital (alpha: 369 (13.6%); wild type: 300 (13.7%)). In fully adjusted analysis, accounting for demographic factors, regional variation, and individual level comorbidities, alpha was associated with 44% increased hazards of death (aHR: 1.44 (95% CI: 1.11 - 1.87); $P$=0.0057) when compared to wild type after conditioning on hospital admission (Figure 2). The increased hazard of death for alpha conditional on hospital admission was consistent across all predefined subgroups and sensitivity analyses (supplementary Figure S2).

Among people admitted to hospital, 615/4,910 (12.5%) were admitted to the intensive care unit (ICU) (alpha: 344; wild type: 271). Compared to people admitted to hospital, those admitted to ICU were of similar age (median age 59.0 vs. 58.0), with a higher proportion having one comorbidity (1 comorbidity: 31.5% vs. 27.4%; 2+ comorbidities: 19.5% vs. 21.9%). Mortality among those admitted to ICU was high (alpha: 147/344 (42.7%); wild type: 99/271 (36.5%)). In fully-adjusted analysis, accounting for demographic factors, regional variation, and individual level comorbidities, the association between alpha and increased mortality was smaller and the confidence interval included the null (aHR: 1.20 (95% CI 0.74 - 1.95; P=0.45), compared to wild type cases after conditioning on admission to the ICU (Figure 2).

### Discussion

This study describes the relative severity of the alpha SARS-CoV-2 variant compared to wild type virus at each stage on the pathway from testing positive to hospital admission and death. The results confirm that alpha causes more severe outcomes, with a 73% increased hazard of death and 62% increased hazard of hospitalisation following a positive test in the community. These findings were consistent across all pre-specified sensitivity analyses, including epidemiological week of infection, meaning they cannot be explained by changing eligibility or external phenomena such as hospitals exceeding capacity. These results are in agreement with previous studies which have shown the alpha variant to be associated with higher case fatality in large populations selected based upon positive tests in the community.[4-6,8,13]

By following people through the pathway of disease, we are able to describe the weakening association between the alpha variant and mortality as the study population is conditioned on more severe disease. When conditioning on hospital admission, alpha was associated with 44% increased hazards of death, when further conditioning on disease severe enough to require admission to ICU there was no evidence that alpha was associated with higher case fatality than wild type virus, although power for this analysis was limited and the confidence interval was consistent with the estimate from the full study population.

Studies among people admitted to ICU may not provide reliable estimates of relative case fatality.

These findings are in agreement with studies that have assessed relative case fatality of the alpha variant among hospitalised patients.[9,14] However, there is no contradiction in the results showing increased mortality for alpha in the community, but no evidence of increased mortality for alpha among those admitted to ICU. Risk factors for death following SARS-CoV-2 infection have been described in detail elsewhere,[15] with the predominant risk factors being older age and the presence of comorbidities. Conditioning on hospital admission controls for these risk factors to some extent, as seen by the older age and higher prevalence of comorbidities among hospitalised patients. Further, people admitted to ICU are a complex study population. In order to be admitted to ICU the attending clinician must consider the illness to be severe enough to require intensive care, but also that the person has a reasonable chance of survival. So people with alpha and wild type virus admitted to ICU may be predisposed to similar case fatality.

It remains the case that even if there is no difference in relative mortality among people with alpha and wild type virus admitted to ICU, a variant that results in more people being admitted to hospital and ICU will have higher case fatality.

In Frampton et al,[9] which studied 341 patients hospitalised with SARS-CoV-2, no association between alpha and increased mortality was found. These findings support the above reasoning as their population was selected on acutely admitted and severely ill patients. Further, although no evidence of a difference in mortality risk was found, the group with the alpha variant had higher viral load, were younger, and had fewer comorbidities, which is consistent with a more severe disease.

The absolute risk estimates for death and hospitalisation following a positive test presented here relate specifically to an unvaccinated population as they are derived from a time when vaccination against SARS-CoV-2 was rare and vaccination prior to infection was an exclusion criterion. These estimates provide important context for assessing the severity of future variants and the impact of the vaccination campaign on the ongoing pandemic. Recent estimates from Public Health England indicate that two doses of Pfizer or Oxford-AstraZeneca vaccine against SARS-CoV-2 reduce the risk of hospitalisation by more than 90%.[16] From our data on the alpha variant, this would result in a reduction of the number of hospitalisations among females under the age of 40 with no comorbidities from 7 in 1000 to 7 in 10,000. Among males over the age of 85 with two or more comorbidities the reduction would be from 39 in 100 to 3.9 in 100.

The strengths of our study include the large study population with individual-level data from primary care on coded diagnoses, medications, vaccinations, and physiological parameters. Linking these data to key datasets, such as ONS deaths data, means we have complete outcome determination for our study period. The main limitation of the analysis is that alpha and wild type viruses are determined by the SGTF proxy, which is less accurate for variant determination than sequencing. However, analysis indicates the sensitivity of SGTF over the study period is over 95%, and previous work has shown that the prevalence of SGTF in the OpenSAFELY study population is representative of England.[6]

SGTF data are available only for people testing positive for SARS-CoV-2 in the community, as a result people with mild or asymptomatic infections who do not present for testing are not included, which may result in overestimation of the absolute risks of death and hospital admission. In addition, SARS-CoV-2 tests performed in hospitals in England are not tested for SGTF, consequently people tested first in hospital, i.e. in emergency departments or on admission, are not included despite being likely to have more severe disease than those tested in the community.

Our study shows that the SARS-CoV-2 alpha variant causes more severe disease than wild type virus following the pathway of illness from test positive to hospital admission and death.

### Acknowledgements
We are grateful for the support received from the TPP Technical Operations team and for generous assistance from the information governance and database teams at NHS England / NHSX.

Funding

This work was jointly funded by UKRI, NIHR and Asthma UK-BLF [COV0076; MR/V015737/] and the Longitudinal Health and Wellbeing strand of the National Core Studies programme. The OpenSAFELY data science platform is funded by the Wellcome Trust. TPP provided technical expertise and infrastructure within their data centre pro bono in the context of a national emergency.

Rosalind Eggo is funded by HDR UK (grant: MR/S003975/1), MRC (grant: MC_PC 19065), NIHR (grant: NIHR200908).

### Conflicts of interest
EW has received payment from AstraZeneca and Roche for providing training and advice, unrelated to the current work. HIM is funded by the National Institute for Health Research (NIHR) Health Protection Research Unit (HPRU) in Vaccines and Immunisation; has been an unpaid invited expert (expert advisory group (EAG)) to the Commission on Human Medicines (CHM) COVID-19 Vaccines Safety Surveillance Methodologies Expert Working Group. LS reports institutional research grants from UKRI, NIHR, and Wellcome, during the conduct of the study. NJD reports grants/support from BMBF, Fetzer Franklin Fund, Good Thinking Society, and Laura and John Arnold Fdn (These are all grants outside the submitted work that should not present any direct conflicts); travel support from Kellogg College and Nuffield Dept. of Primary Care Health Sciences (These are their department and college at Oxford and should not represent any conflict with this work). RM reports grants/contracts/support from Wellcome Trust, serving on an advisory board/DSMB for Wellcome Trust, and receiving consulting fees from AMGEN, outside the submitted work. RME reports grants from UKRI and HDR UK (All standard Funding agency grants to institution), during the conduct of the study; grants from IMI2 (Standard funding agency grant to institution), outside the submitted work. BG reports support from Wellcome, MRC/NIHR, and NCS LHW (OpenSAFELY platform grants), during the conduct of the study; BG has received research funding from the Laura and John Arnold Foundation, the NHS National Institute for Health Research (NIHR), the NIHR School of Primary Care Research, the NIHR Oxford Biomedical Research Centre, the Mohn-Westlake Foundation, NIHR Applied Research Collaboration Oxford and Thames Valley, the Wellcome Trust, the Good Thinking Foundation, Health Data Research UK, the Health Foundation, the World Health Organisation, UKRI, Asthma UK, the British Lung Foundation, and the Longitudinal Health and Wellbeing strand of the National Core Studies programme, outside the submitted work. BG reports personal income from journalism, public speaking and pop science books on the misuse of science. BG reports the following leadership roles: UK Gov, DHSC HealthTEch Advisory Board, leading review for Sec of State, NED at NHS Digital. KB reports a fellowship with Wellcome, payment to institution, during the conduct of the study. AS reports being employed by LSHTM on a fellowship sponsored by GSK outside of the submitted work. IJD reports grants to LSHTM from GSK and GSK shares outside of the submitted work. LT reports grants or contracts from Wellcome, MRC, and NIHR; support for MHRA Expert Advisory Group x 4; member of 2 non-industry funded trial advisory committees (unpaid) all outside of the submitted work.

Table 1 Absolute risk of death by 28-days following positive test for SARS-CoV-2, expressed as a percentage

| Comorbidities/Sex/Age group | Wild type<br>% (95% CI) | Alpha<br>% (95% CI) |
|---|---|---|
| **No Comorbidities** | | |
| **Female: 0-<40** | 0.01 (0.00-0.01) | 0.01 (0.00-0.02) |
| **40-<55** | 0.06 (0.04-0.08) | 0.10 (0.07-0.13) |
| **55-<65** | 0.17 (0.12-0.23) | 0.29 (0.21-0.38) |
| **65-<75** | 0.64 (0.45-0.83) | 1.07 (0.77-1.37) |
| **75-<85** | 1.58 (1.10-2.07) | 2.63 (1.85-3.41) |
| **85+** | 3.69 (2.44-4.93) | 6.03 (4.04-8.01) |
| **Male: 0-<40** | 0.02 (0.00-0.03) | 0.03 (0.01-0.04) |
| **40-<55** | 0.11 (0.07-0.14) | 0.18 (0.12-0.24) |
| **55-<65** | 0.32 (0.22-0.41) | 0.54 (0.39-0.68) |
| **65-<75** | 1.16 (0.84-1.49) | 1.94 (1.43-2.44) |
| **75-<85** | 2.85 (1.99-3.71) | 4.68 (3.34-6.03) |
| **85+** | 6.49 (4.31-8.67) | 10.40 (7.08-13.72) |
| **1 Comorbidity** | | |
| **Female: 0-<40** | 0.01 (0.00-0.02) | 0.02 (0.01-0.04) |
| **40-<55** | 0.09 (0.06-0.13) | 0.15 (0.10-0.21) |
| **55-<65** | 0.27 (0.18-0.36) | 0.46 (0.31-0.60) |
| **65-<75** | 1.00 (0.72-1.28) | 1.66 (1.21-2.11) |
| **75-<85** | 2.45 (1.77-3.13) | 4.04 (2.94-5.13) |
| **85+** | 5.61 (3.94-7.29) | 9.05 (6.41-11.68) |
| **Male: 0-<40** | 0.02 (0.01-0.04) | 0.04 (0.01-0.07) |
| **40-<55** | 0.17 (0.11-0.23) | 0.28 (0.18-0.38) |
| **55-<65** | 0.50 (0.35-0.65) | 0.83 (0.59-1.08) |
| **65-<75** | 1.80 (1.33-2.28) | 2.99 (2.25-3.73) |
| **75-<85** | 4.36 (3.20-5.53) | 7.09 (5.29-8.90) |
| **85+** | 9.70 (6.88-12.52) | 15.21 (11.04-19.38) |
| **2+ Comorbidities** | | |
| **Female: 0-<40** | 0.02 (0.01-0.04) | 0.04 (0.01-0.07) |
| **40-<55** | 0.17 (0.11-0.24) | 0.29 (0.18-0.41) |
| **55-<65** | 0.52 (0.34-0.69) | 0.86 (0.58-1.14) |
| **65-<75** | 1.87 (1.35-2.38) | 3.09 (2.26-3.92) |
| **75-<85** | 4.51 (3.39-5.63) | 7.33 (5.56-9.09) |
| **85+** | 10.01 (7.47-12.55) | 15.66 (11.85-19.47) |
| **Male: 0-<40** | 0.04 (0.01-0.08) | 0.08 (0.02-0.13) |
| **40-<55** | 0.32 (0.20-0.44) | 0.53 (0.33-0.73) |
| **55-<65** | 0.94 (0.64-1.23) | 1.57 (1.09-2.04) |
| **65-<75** | 3.35 (2.50-4.19) | 5.48 (4.17-6.79) |
| **75-<85** | 7.88 (6.07-9.68) | 12.50 (9.80-15.20) |
| **85+** | 16.65 (12.70-20.60) | 24.97 (19.53-30.41) |

Table 2 Absolute risk of hospitalisation by 28-days following positive test for SARS-CoV-2, expressed as a percentage

| Comorbidities/Sex/Age group | Wild type (95% CI) | Alpha (95% CI) |
|---|---|---|
| **No Comorbidities** | | |
| **Female: 0-<40** | 0.55 (0.49-0.61) | 0.74 (0.67-0.82) |
| **40-<55** | 1.45 (1.32-1.59) | 1.96 (1.79-2.12) |
| **55-<65** | 2.26 (2.03-2.48) | 3.02 (2.73-3.32) |
| **65-<75** | 4.16 (3.70-4.63) | 5.54 (4.93-6.14) |
| **75-<85** | 5.76 (4.95-6.57) | 7.61 (6.56-8.65) |
| **85+** | 9.82 (7.97-11.67) | 12.77 (10.44-15.09) |
| **Male: 0-<40** | 0.84 (0.75-0.92) | 1.13 (1.01-1.24) |
| **40-<55** | 2.20 (2.01-2.39) | 2.95 (2.71-3.19) |
| **55-<65** | 3.39 (3.06-3.72) | 4.52 (4.11-4.94) |
| **65-<75** | 6.19 (5.51-6.86) | 8.16 (7.31-9.00) |
| **75-<85** | 8.47 (7.31-9.64) | 11.07 (9.60-12.54) |
| **85+** | 14.11 (11.55-16.68) | 18.07 (14.94-21.20) |
| **1 Comorbidity** | | |
| **Female: 0-<40** | 1.02 (0.88-1.15) | 1.37 (1.19-1.55) |
| **40-<55** | 2.67 (2.38-2.95) | 3.57 (3.20-3.94) |
| **55-<65** | 4.10 (3.67-4.53) | 5.45 (4.90-6.01) |
| **65-<75** | 7.43 (6.63-8.22) | 9.74 (8.73-10.76) |
| **75-<85** | 10.11 (8.82-11.41) | 13.13 (11.50-14.77) |
| **85+** | 16.62 (13.84-19.40) | 21.09 (17.73-24.46) |
| **Male: 0-<40** | 1.54 (1.35-1.74) | 2.08 (1.81-2.34) |
| **40-<55** | 4.00 (3.59-4.41) | 5.32 (4.80-5.84) |
| **55-<65** | 6.10 (5.50-6.70) | 8.04 (7.29-8.79) |
| **65-<75** | 10.82 (9.74-11.90) | 14.02 (12.69-15.35) |
| **75-<85** | 14.51 (12.75-16.28) | 18.55 (16.40-20.70) |
| **85+** | 23.05 (19.46-26.65) | 28.64 (24.47-32.81) |
| **2+ Comorbidities** | | |
| **Female: 0-<40** | 1.63 (1.39-1.87) | 2.19 (1.87-2.51) |
| **40-<55** | 4.21 (3.68-4.75) | 5.60 (4.90-6.30) |
| **55-<65** | 6.41 (5.64-7.18) | 8.44 (7.46-9.43) |
| **65-<75** | 11.34 (10.10-12.59) | 14.67 (13.11-16.22) |
| **75-<85** | 15.18 (13.41-16.95) | 19.36 (17.18-21.54) |
| **85+** | 23.99 (20.52-27.46) | 29.72 (25.68-33.75) |
| **Male: 0-<40** | 2.46 (2.10-2.81) | 3.29 (2.83-3.76) |
| **40-<55** | 6.26 (5.51-7.01) | 8.25 (7.29-9.20) |
| **55-<65** | 9.40 (8.36-10.43) | 12.24 (10.94-13.53) |
| **65-<75** | 16.17 (14.56-17.78) | 20.55 (18.61-22.50) |
| **75-<85** | 21.21 (18.93-23.48) | 26.51 (23.82-29.19) |
| **85+** | 32.12 (27.93-36.31) | 38.79 (34.16-43.41) |

Figure 1 Summary population characteristics for alpha and wild type infections a) Regional distribution of alpha cases; b) number of alpha and wild type cases by epidemiological week; c) number of outcomes analysed; d) age distribution (Median, IQR); e) presence of comorbidities

Figure 2 Relative severity of alpha compared to wild type virus aHR: adjusted hazard ratio; ICU: intensive care unit; Death | hospital admission: death given hospital admission; Death | ICU: death given ICU admission.

All models include covariate adjustment for age, sex, ethnicity, smoking status, obesity status, categorical number of comorbidities, index of multiple deprivation, household size, residential rural or urban location classification, epidemiological week, and care home status. Except for the death given ICU admission model, which excludes adjustment for care home status.

Cox proportional hazards regression; all models are stratified on region by UTLA; estimating a separate baseline hazard function for each UTLA, with model parameters estimated by maximum likelihood over the full study population.

Figure 3 Summary characteristics of deaths occurring with and without hospital admission a) total deaths; b) age distribution (Median, IQR); c) presence of comorbidities; d) sex and care home residence proportions.

Figure 1

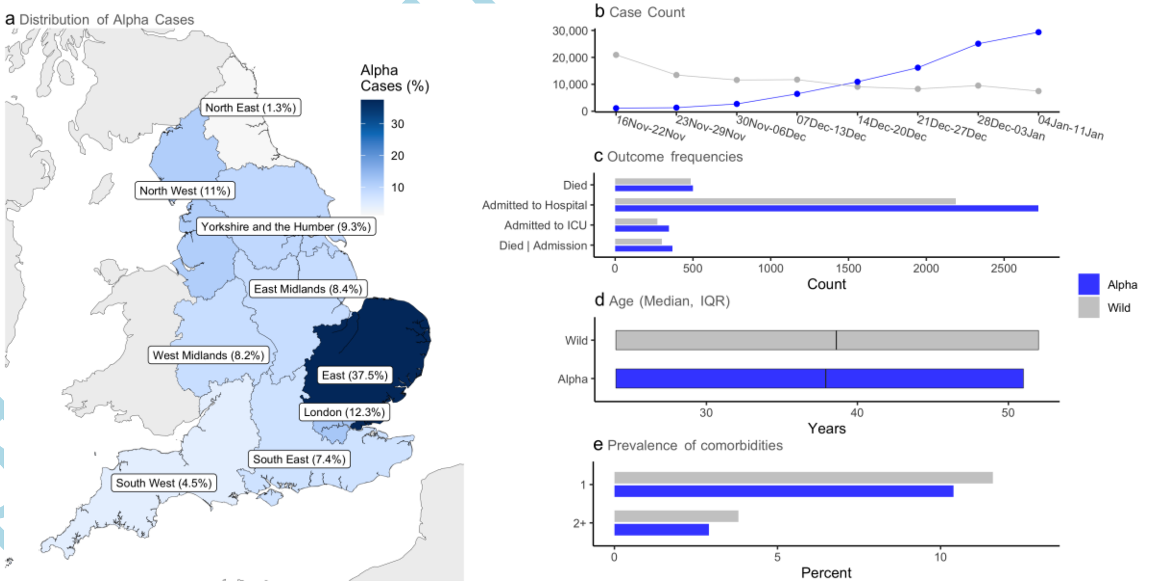

Figure 2

Outcome
N (Events)
Alpha
Wild type
aHR for Alpha vs. Wild type
aHR (95% CI; P-value)
Death
60,813 (350)
60,831 (328)
1.73 (1.41 - 2.13; <.0001)
Hospital admission
60,813 (1,856)
60,831 (1,572)
1.62 (1.48 - 1.78; <.0001)
Death | Hospital admission
1,856 (259)
1,572 (204)
1.44 (1.11 - 1.87; 0.0057)
Death | ICU admission
245 (102)
191 (68)
1.20 (0.74 - 1.95; 0.45)
Increased hazards with Alpha →
0.75
1
1.25
1.5
1.75
2
2.25

Figure 3

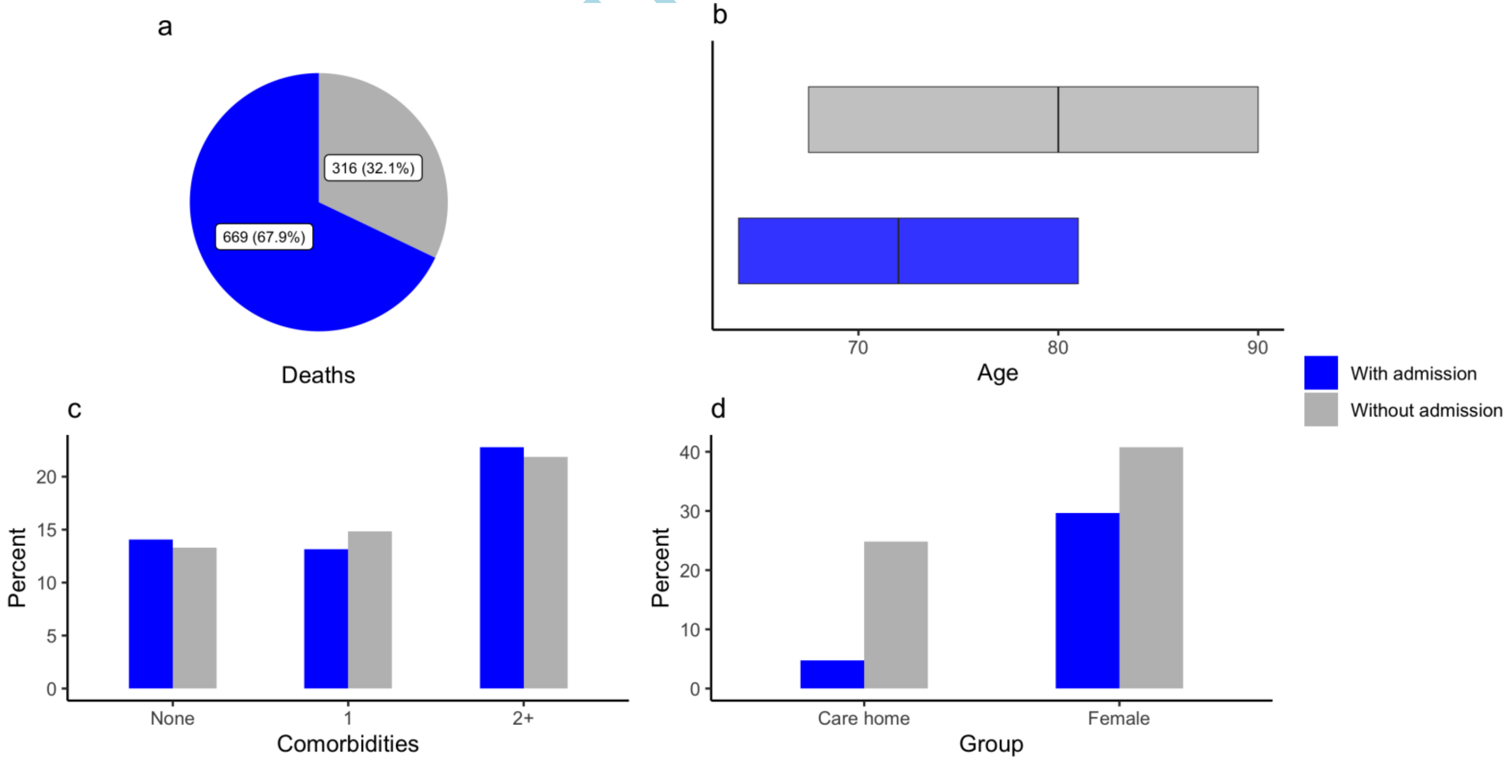

a
316 (32.1%)
669 (67.9%)
Deaths
b
70
80
90
Age
With admission
Without admission
c
Percent
0
5
10
15
20
None
1
2+
Comorbidities
d
Percent
0
10
20
30
40
Care home
Female
Group

## Supplementary Material

ciab754_suppl_Supplementary_Materials

