# Severity of Severe Acute Respiratory System Coronavirus 2 (SARS-CoV-2) Alpha Variant (B.1.1.7) in England

**DOI:** 10.1093/cid/ciab754

**Published:** 2021-09-06

**Authors:** Daniel J Grint, Kevin Wing, Catherine Houlihan, Hamish P Gibbs, Stephen J W Evans, Elizabeth Williamson, Helen I McDonald, Krishnan Bhaskaran, David Evans, Alex J Walker, George Hickman, Emily Nightingale, Anna Schultze, Christopher T Rentsch, Chris Bates, Jonathan Cockburn, Helen J Curtis, Caroline E Morton, Sebastian Bacon, Simon Davy, Angel Y S Wong, Amir Mehrkar, Laurie Tomlinson, Ian J Douglas, Rohini Mathur, Brian MacKenna, Peter Ingelsby, Richard Croker, John Parry, Frank Hester, Sam Harper, Nicholas J DeVito, Will Hulme, John Tazare, Liam Smeeth, Ben Goldacre, Rosalind M Eggo

**Affiliations:** Faculty of Epidemiology and Population Health, London School of Hygiene and Tropical Medicine, London, United Kingdom; Faculty of Epidemiology and Population Health, London School of Hygiene and Tropical Medicine, London, United Kingdom; Division of Infection and Immunity, University College London, London, United Kingdom; Faculty of Epidemiology and Population Health, London School of Hygiene and Tropical Medicine, London, United Kingdom; Faculty of Epidemiology and Population Health, London School of Hygiene and Tropical Medicine, London, United Kingdom; Faculty of Epidemiology and Population Health, London School of Hygiene and Tropical Medicine, London, United Kingdom; Faculty of Epidemiology and Population Health, London School of Hygiene and Tropical Medicine, London, United Kingdom; Faculty of Epidemiology and Population Health, London School of Hygiene and Tropical Medicine, London, United Kingdom; The DataLab, Nuffield Department of Primary Care Health Sciences, University of Oxford, Oxford, United Kingdom; The DataLab, Nuffield Department of Primary Care Health Sciences, University of Oxford, Oxford, United Kingdom; The DataLab, Nuffield Department of Primary Care Health Sciences, University of Oxford, Oxford, United Kingdom; Faculty of Public Health and Policy, London School of Hygiene and Tropical Medicine, London, United Kingdom; Faculty of Epidemiology and Population Health, London School of Hygiene and Tropical Medicine, London, United Kingdom; Faculty of Epidemiology and Population Health, London School of Hygiene and Tropical Medicine, London, United Kingdom; TPP, Horsforth, Leeds, United Kingdom; TPP, Horsforth, Leeds, United Kingdom; The DataLab, Nuffield Department of Primary Care Health Sciences, University of Oxford, Oxford, United Kingdom; The DataLab, Nuffield Department of Primary Care Health Sciences, University of Oxford, Oxford, United Kingdom; The DataLab, Nuffield Department of Primary Care Health Sciences, University of Oxford, Oxford, United Kingdom; The DataLab, Nuffield Department of Primary Care Health Sciences, University of Oxford, Oxford, United Kingdom; Faculty of Epidemiology and Population Health, London School of Hygiene and Tropical Medicine, London, United Kingdom; The DataLab, Nuffield Department of Primary Care Health Sciences, University of Oxford, Oxford, United Kingdom; Faculty of Epidemiology and Population Health, London School of Hygiene and Tropical Medicine, London, United Kingdom; Faculty of Epidemiology and Population Health, London School of Hygiene and Tropical Medicine, London, United Kingdom; Faculty of Epidemiology and Population Health, London School of Hygiene and Tropical Medicine, London, United Kingdom; The DataLab, Nuffield Department of Primary Care Health Sciences, University of Oxford, Oxford, United Kingdom; The DataLab, Nuffield Department of Primary Care Health Sciences, University of Oxford, Oxford, United Kingdom; The DataLab, Nuffield Department of Primary Care Health Sciences, University of Oxford, Oxford, United Kingdom; TPP, Horsforth, Leeds, United Kingdom; TPP, Horsforth, Leeds, United Kingdom; TPP, Horsforth, Leeds, United Kingdom; The DataLab, Nuffield Department of Primary Care Health Sciences, University of Oxford, Oxford, United Kingdom; The DataLab, Nuffield Department of Primary Care Health Sciences, University of Oxford, Oxford, United Kingdom; Faculty of Epidemiology and Population Health, London School of Hygiene and Tropical Medicine, London, United Kingdom; Faculty of Epidemiology and Population Health, London School of Hygiene and Tropical Medicine, London, United Kingdom; The DataLab, Nuffield Department of Primary Care Health Sciences, University of Oxford, Oxford, United Kingdom; Faculty of Epidemiology and Population Health, London School of Hygiene and Tropical Medicine, London, United Kingdom

**Keywords:** SARS-CoV-2, alpha, case fatality, hospital admission

## Abstract

**Background:**

The severe acute respiratory syndrome coronavirus 2 (SARS-CoV-2) alpha variant (B.1.1.7) is associated with higher transmissibility than wild-type virus, becoming the dominant variant in England by January 2021. We aimed to describe the severity of the alpha variant in terms of the pathway of disease from testing positive to hospital admission and death.

**Methods:**

With the approval of NHS England, we linked individual-level data from primary care with SARS-CoV-2 community testing, hospital admission, and Office for National Statistics all-cause death data. We used testing data with S-gene target failure as a proxy for distinguishing alpha and wild-type cases, and stratified Cox proportional hazards regression to compare the relative severity of alpha cases with wild-type diagnosed from 16 November 2020 to 11 January 2021.

**Results:**

Using data from 185 234 people who tested positive for SARS-CoV-2 in the community (alpha = 93 153; wild-type = 92 081), in fully adjusted analysis accounting for individual-level demographics and comorbidities as well as regional variation in infection incidence, we found alpha associated with 73% higher hazards of all-cause death (adjusted hazard ratio [aHR]: 1.73; 95% confidence interval [CI]: 1.41–2.13; *P* < .0001) and 62% higher hazards of hospital admission (1.62; 1.48–1.78; *P* < .0001) compared with wild-type virus. Among patients already admitted to the intensive care unit, the association between alpha and increased all-cause mortality was smaller and the CI included the null (aHR: 1.20; 95% CI: .74–1.95; *P* = .45).

**Conclusions:**

The SARS-CoV-2 alpha variant is associated with an increased risk of both hospitalization and mortality than wild-type virus.

## Teaser

The SARS-CoV-2 alpha variant is associated with a 62% increased risk of hospitalization and a 73% increased risk of death, compared with the originally circulating wild-type virus in England between 16 November 2020 and 21 April 2021.

The severe acute respiratory syndrome coronavirus 2 (SARS-CoV-2; coronavirus disease 2019 [COVID-19]) variant of concern B.1.1.7, now called the alpha variant (alpha), was first identified in Kent, United Kingdom, in autumn 2020 [1]. Early analysis estimated that alpha is more transmissible than the original lineage and it became the dominant strain throughout the United Kingdom in early 2021 [2]. Only a small number of alpha cases were originally identified by whole-genome sequencing. Certain polymerase chain reaction (PCR) assays for SARS-CoV-2 that are used in 3 major laboratories in England do not amplify one of the spike protein gene targets in the alpha variant. Spike gene target failure (SGTF) was therefore adopted as a proxy for identifying alpha and has been shown to have more than 95% sensitivity for alpha viruses during the period 16 November 2020 to 11 January 2021 [3].

While a number of studies have shown that alpha is associated with an overall higher case fatality than the original lineage [4–8], studies specifically restricted to hospitalized patients have shown no difference in case fatality [8, 9]. However, these findings are not necessarily contradictory as alpha may cause more severe disease, leading to more people needing hospital admission, but may not be any more likely than the original lineage to cause death in those who already have severe disease requiring hospital care.

This study aims to bring these elements together in a consolidated analysis, following the pathway of disease from infection to hospital admission and death, in order to fully illuminate the association of the alpha variant with altered healthcare need and mortality.

## METHODS

### Data Platform

With the approval of NHS England, data were linked, stored, and analyzed securely within the OpenSAFELY electronic health records research platform [10]. OpenSAFELY holds electronic health records (EHRs) for 58 million individual registrations with a general practitioner (GP) in England, and in this study we use a subset of these who are registered at practices using the TPP EHR management system, which includes 24 million people, covering 40% of England’s population. Primary care data include individual-level coded diagnoses, medications, vaccinations, and physiological parameters. These data were linked to key datasets to obtain the following: (1) SARS-CoV-2 community testing data through the Second Generation Surveillance System, (2) hospital admission data, (3) COVID-19–related intensive care unit (ICU) admission data, and (4) all-cause registered deaths from the Office for National Statistics (ONS). More information on the OpenSAFELY analytical platform and data sources is available in sections 1–3 in the Supplementary Material (Supplementary Table 1).

### Study Design and Population

We defined our study population as all who tested positive for SARS-CoV-2 in the community with data available on SGTF status, between 16 November 2020 and 11 January 2021. During this time, alpha cases increased from a small minority as a proportion of all diagnosed SARS-CoV-2 infections in the United Kingdom to the dominant majority. This period of crossover from the original lineage to alpha presents the ideal cohort for comparison of the relative severity of alpha compared with wild-type virus. The study period predates the emergence of the delta variant.

The primary exposure of interest was SGTF status. Spike gene target failure was taken as a proxy for identifying the SARS-CoV-2 alpha variant, and compared with cases without S-gene dropout (wild-type).

### Statistical Methods

The primary analysis used a Cox proportional hazards regression model stratified by geographic region, defined as the upper tier local authority area (UTLA) [11, 12]. Stratification by region allowed a separate hazard function to be estimated for each region, with parameter estimates estimated over the full population. This degree of regional flexibility was included a priori to account for potentially nonproportional changes in pandemic incidence over time by region.

For analysis of all-cause mortality, follow-up began at the date of testing positive in the community for SARS-CoV-2 and was censored at 21 April 2021 or 7 days prior to receipt of a vaccination against SARS-CoV-2. Since illness that may lead to death would exclude the booking and administration of a vaccine, the 7 days prior to vaccination were censored to discount a potential immortal time bias.

For analysis of hospital admission, follow-up began at the date of testing positive for SARS-CoV-2 and was censored at 21 April 2021, the date of deregistration from GP practice, or 7 days prior to receipt of vaccination against SARS-CoV-2.

For analysis of all-cause mortality among those admitted to a hospital, follow-up began at the date of hospital admission and was censored at 21 April 2021 or 7 days prior to receipt of vaccination against SARS-CoV-2. In England, the National Health Service (NHS) vaccination program for SARS-CoV-2 began in December 2020; consequently, censoring on vaccination was rare in this study population. A further analysis of those admitted to a hospital was performed on the population who spent time in the ICU during their hospital stay. This subset further conditions the population admitted to a hospital to be those with severe illness who received intensive care.

Covariate adjustment was informed by consideration of causal pathways using a causal diagram. Subgroup analysis of the primary exposure was prespecified for epidemiological week of SARS-CoV-2 diagnosis, comorbidity status, ethnicity in 5 categories, deprivation quintile, and age group.

Comorbidities were defined as in our prior work [6] as the presence of codes in the patient’s EHR indicating diagnoses. All codes and conditions are given in section 4 of the Supplementary Material (Supplementary Table 2).

A number of prespecified sensitivity analyses were also performed including censoring all follow-up 28 days after SARS-CoV-2 diagnosis, restricting to the population with a minimum of 40 days’ follow-up, and imputing missing data on ethnicity. Further information on analysis methods and full details of all prespecified sensitivity and subgroup analyses are available in the study protocol (https://github.com/opensafely/SGTF-CFR-research/tree/master/docs/).

Absolute risk estimates were calculated from the marginal means of fully adjusted logistic regression models with the outcomes of death by 28 days after a positive SARS-CoV-2 test and hospital admission by 28 days after a positive SARS-CoV-2 test. In each case, the population was restricted to those who had a minimum of 28 days’ follow-up from the date of their positive SARS-CoV-2 test to the follow-up censor. In these models, deaths and hospital admissions beyond 28 days were censored. Vaccination prior to SARS-CoV-2 infection was an exclusion criterion in this analysis.

## RESULTS

### Population Characteristics

**Figure 1.**
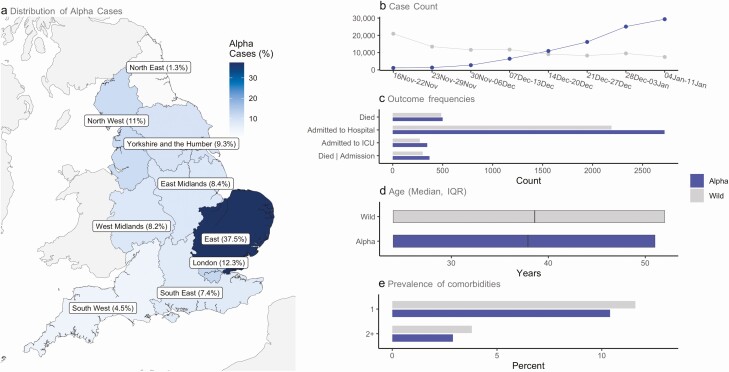
Summary population characteristics for alpha and wild-type infections. *a*, Regional distribution of alpha cases; b, number of alpha and wild-type cases by epidemiological week; *c*, number of outcomes analyzed; *d*, age distribution (median, IQR); *e*, presence of comorbidities. Abbreviations: ICU, intensive care unit; IQR, interquartile range.

Our study population consists of 185 234 people testing positive for SARS-CoV-2 in the community between 16 November 2020 and 11 January 2021 for whom SGTF status was known (alpha = 93 153; wild-type = 92 081). In the week beginning 16 November 2020 wild-type cases accounted for 20 926 of 22 062 (94.9%) total cases; by the week beginning 4 January, 29, 349 of 36 821 (79.7%) cases were alpha. Consequently, median follow-up time was shorter for alpha cases (102.0 [interquartile range (IQR): 62.0–113.0] days vs 109.0 [71.0–136.0] days) compared with wild-type cases. Overall, alpha cases were concentrated in the East (37.5%), London (12.3%), and North West (11.0%), reflecting areas where the alpha epidemic began. Wild-type cases were mainly from Yorkshire and the Humber (25.3%), the East Midlands (20.3%), and North West (16.0%) (Figure 1; Supplementary Table 3).

People with the alpha variant were marginally younger overall (median age: 37.0 vs 38.0 years), with a smaller proportion of people aged 70 to less than 80 years (2.9% vs 3.4%) and 80 years and older (0.9% vs 1.7%), compared with wild-type cases. Fewer people infected with the alpha variant had underlying comorbidities (1 comorbidity [10.4% vs 11.6%]; ≥2 comorbidities [2.9% vs 3.8%]) compared with those infected with wild-type virus. The proportion of people identified as living in care homes was lower for alpha cases (0.1% vs 0.4%). A lower proportion of people with the alpha variant lived in areas of the most deprived socioeconomic status (SES) quintile (16.7% vs 26.3%), whereas a higher proportion lived in areas of the least-deprived SES quintile (22.1% vs 17.4%), compared with people with wild-type virus (Figure 1; Supplementary Table 3).

### Case Fatality

**Figure 2.**
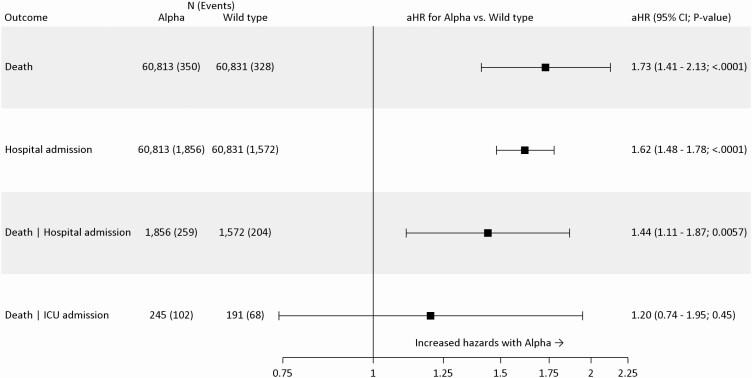
Relative severity of alpha compared with wild-type virus. All models include covariate adjustment for age, sex, ethnicity, smoking status, obesity status, categorical number of comorbidities, index of multiple deprivation, household size, residential rural or urban location classification, epidemiological week, and care home status, except for the Death | ICU admission model, which excludes adjustment for care home status. Cox proportional hazards regression was used; all models are stratified on region by UTLA, estimating a separate baseline hazard function for each UTLA, with model parameters estimated by maximum likelihood over the full study population. Abbreviations: aHR, adjusted hazard ratio; CI, confidence interval; Death | Hospital admission, death given hospital admission; Death | ICU admission, death given ICU admission; ICU, intensive care unit; UTLA, upper tier local authority area.

A total of 985 deaths of any cause were registered by 21 April 2021 (alpha: 500 [0.5%]; wild-type: 485 [0.5%]). In fully adjusted analysis, accounting for demographic factors, regional variation, and individual-level comorbidities, alpha was associated with 73% increased hazards of death (adjusted hazard ratio [aHR]: 1.73; 95% confidence interval [CI]: 1.41–2.13; *P* < .0001) when compared with wild-type (Figure 2). The increased hazard of death for alpha was consistent across all predefined subgroups and sensitivity analyses (Supplementary Figure 1).

The absolute risk of death by 28 days post–positive SARS-CoV-2 test for people with alpha was low for males (.03%; 95% CI: .01–.04%) and females (.01%; .00–.2%) aged 40 years and younger in the absence of comorbidities. However, the risk of death by 28 days was considerable for males (10.4% [7.1–13.7%]) and females (6.0% [4.0–8.0%]) aged 85 years and older with alpha. In the presence of 2 or more comorbidities, the risk of death by 28 days for those with alpha was increased for males (.08%; 95% CI: .02–.13%) and females (.04%; 95% CI: .01–.07%) aged 40 and younger, and was particularly high for males (25.0%; 95% CI: 19.5–30.4%) and females (15.7%; 95% CI: 11.9–19.5%) aged 85 and older (Table 1).

**Table 1.** Absolute Risk of Death by 28 Days Following a Positive Test for SARS-CoV-2, Expressed as a Percentage

	% (95% CI)	
Comorbidities/Sex/Age Group	Wild-type	Alpha
No comorbidities		
Females		
0 to <40 years	.01 (.00–.01)	.01 (.00–.02)
40 to <55 years	.06 (.04–.08)	.10 (.07–.13)
55 to <65 years	.17 (.12–.23)	.29 (.21–.38)
65 to <75 years	.64 (.45–.83)	1.07 (.77–1.37)
75 to <85 years	1.58 (1.10–2.07)	2.63 (1.85–3.41)
≥85 years	3.69 (2.44–4.93)	6.03 (4.04–8.01)
Males		
0 to <40 years	.02 (.00–.03)	.03 (.01–.04)
40 to <55 years	.11 (.07–.14)	.18 (.12–.24)
55 to <65 years	.32 (.22–.41)	.54 (.39–.68)
65 to <75 years	1.16 (0.84–1.49)	1.94 (1.43–2.44)
75 to <85 years	2.85 (1.99–3.71)	4.68 (3.34–6.03)
≥85 years	6.49 (4.31–8.67)	10.40 (7.08–13.72)
One comorbidity		
Females		
0 to <40 years	.01 (.00–.02)	.02 (.01–.04)
40 to <55 years	.09 (.06–.13)	.15 (.10–.21)
55 to <65 years	.27 (.18–.36)	.46 (.31–.60)
65 to <75 years	1.00 (.72–1.28)	1.66 (1.21–2.11)
75 to <85 years	2.45 (1.77–3.13)	4.04 (2.94–5.13)
≥85 years	5.61 (3.94–7.29)	9.05 (6.41–11.68)
Males		
0 to <40 years	.02 (.01–.04)	.04 (.01–.07)
40 to <55 years	.17 (.11–.23)	.28 (.18–.38)
55 to <65 years	.50 (.35–.65)	.83 (.59–1.08)
65 to <75 years	1.80 (1.33–2.28)	2.99 (2.25–3.73)
75 to <85 years	4.36 (3.20–5.53)	7.09 (5.29–8.90)
≥85 years	9.70 (6.88–12.52)	15.21 (11.04–19.38)
Two or more comorbidities		
Females		
0 to <40 years	.02 (.01–.04)	.04 (.01–.07)
40 to <55 years	.17 (.11–.24)	.29 (.18–.41)
55 to <65 years	.52 (.34–.69)	.86 (.58–1.14)
65 to <75 years	1.87 (1.35–2.38)	3.09 (2.26–3.92)
75 to <85 years	4.51 (3.39–5.63)	7.33 (5.56–9.09)
≥85 years	10.01 (7.47–12.55)	15.66 (11.85–19.47)
Males		
0 to <40 years	.04 (.01–.08)	.08 (.02–.13)
40 to <55 years	.32 (.20–.44)	.53 (.33–.73)
55 to <65 years	.94 (.64–1.23)	1.57 (1.09–2.04)
65 to <75 years	3.35 (2.50–4.19)	5.48 (4.17–6.79)
75 to <85 years	7.88 (6.07–9.68)	12.50 (9.80–15.20)
≥85 years	16.65 (12.70–20.60)	24.97 (19.53–30.41)

Abbreviations: CI, confidence interval; SARS-CoV-2, severe acute respiratory syndrome coronavirus 2.

### Admission to a Hospital

**Figure 3.**
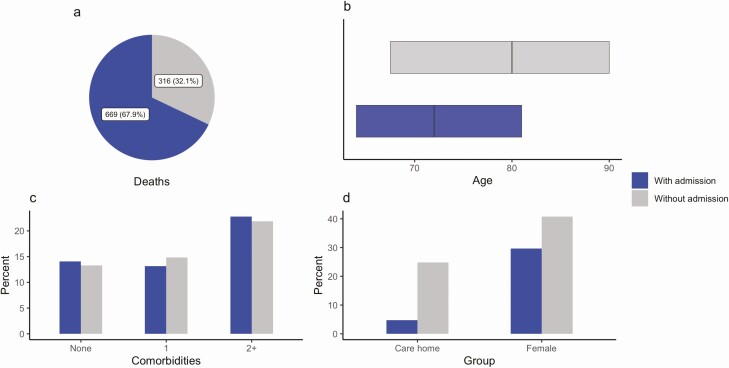
Summary characteristics of deaths occurring with and without hospital admission. *a*, Total deaths; *b*, age distribution (median, IQR); *c*, presence of comorbidities; *d*, sex and care home residence proportions. Abbreviations: ICU, intensive care unit; IQR, interquartile range.

A total of 316 of 985 (32.1%) deaths registered in the study occurred without admission to a hospital (alpha: 131; wild-type: 185). People who died without hospital admission were older (median [IQR] age: 80.0 [67.5–90.0] vs 72.0 [62.0–81.0] years), a higher proportion were female (51.9% vs 37.7%), and a higher proportion were resident in a care home (31.6% vs 6.0%) compared with deaths following admission to a hospital (Figure 3).

A total of 4910 people were admitted to a hospital following a positive test for SARS-CoV-2 in our dataset (alpha: 2721 [2.9%]; wild-type: 2189 [2.4%]). Compared with the full study population, those admitted to a hospital were older (median age: 58.0 vs 38.0 years), with more comorbidities (1 comorbidity: 27.4% vs 11.0%; ≥2 comorbidities: 21.9% vs 3.4%). Among people admitted to a hospital, those with alpha were younger (median [IQR] age: 57.0 [47.0–68.0] vs 59.0 [48.0–72.0] years), and had fewer comorbidities (≥2 comorbidities: 19.3% vs 25.2%) compared with those with wild-type (Supplementary Table 4).

In fully adjusted analysis, accounting for demographic factors, regional variation, and individual-level comorbidities, alpha was associated with 62% increased hazards of hospital admission (aHR: 1.62; 95% CI: 1.48–1.78; *P* < .0001) when compared with wild-type (Figure 2). The increased hazard of hospital admission for alpha was consistent across all predefined subgroups and sensitivity analyses (Supplementary Figure 2).

The absolute risk of hospital admission by 28 day post–positive SARS-CoV-2 test for those with alpha was 1.1% for males (1.1%; 95% CI: 1.01–1.24) and 0.7% for females (.74%; .67–.82%) aged 40 and younger in the absence of comorbidities. However, the risk of hospitalization was considerable for males (18.1%; 14.9-21.2%) and females (12.8%; 10.4–15.1%) aged 85 years and older. In the presence of 2 or more comorbidities the risk of hospital admission for those with alpha was increased for males (3.3%; 95% CI: 2.8–3.8%) and females (2.2%; 1.9–2.5%) aged 40 and younger, and high for males (38.8%; 34.2–43.4%) and females (29.7%; 25.7–33.8%) aged 85 and older (Table 2).

**Table 2.** Absolute Risk of Hospitalization by 28 Days Following a Positive Test for SARS-CoV-2, Expressed as a Percentage

	% (95% CI)	
Comorbidities/Sex/Age group	Wild-type	Alpha
No comorbidities		
Females		
0 to <40 years	.55 (.49–.61)	.74 (.67–.82)
40 to <55 years	1.45 (1.32–1.59)	1.96 (1.79–2.12)
55 to <65 years	2.26 (2.03–2.48)	3.02 (2.73–3.32)
65 to <75 years	4.16 (3.70–4.63)	5.54 (4.93–6.14)
75 to <85 years	5.76 (4.95–6.57)	7.61 (6.56–8.65)
≥85 years	9.82 (7.97–11.67)	12.77 (10.44–15.09)
Males		
0 to <40 years	.84 (.75–.92)	1.13 (1.01–1.24)
40 to <55 years	2.20 (2.01–2.39)	2.95 (2.71–3.19)
55 to <65 years	3.39 (3.06–3.72)	4.52 (4.11–4.94)
65 to <75 years	6.19 (5.51–6.86)	8.16 (7.31–9.00)
75 to <85 years	8.47 (7.31–9.64)	11.07 (9.60–12.54)
≥85 years	14.11 (11.55–16.68)	18.07 (14.94–21.20)
One comorbidity		
Females		
0 to <40 years	1.02 (.88–1.15)	1.37 (1.19–1.55)
40 to <55 years	2.67 (2.38–2.95)	3.57 (3.20–3.94)
55 to <65 years	4.10 (3.67–4.53)	5.45 (4.90–6.01)
65 to <75 years	7.43 (6.63–8.22)	9.74 (8.73–10.76)
75 to <85 years	10.11 (8.82–11.41)	13.13 (11.50–14.77)
≥85 years	16.62 (13.84–19.40)	21.09 (17.73–24.46)
Males		
0 to <40 years	1.54 (1.35–1.74)	2.08 (1.81–2.34)
40 to <55 years	4.00 (3.59–4.41)	5.32 (4.80–5.84)
55 to <65 years	6.10 (5.50–6.70)	8.04 (7.29–8.79)
65 to <75 years	10.82 (9.74–11.90)	14.02 (12.69–15.35)
75 to <85 years	14.51 (12.75–16.28)	18.55 (16.40–20.70)
≥85 years	23.05 (19.46–26.65)	28.64 (24.47–32.81)
Two or more comorbidities		
Females		
0 to <40 years	1.63 (1.39–1.87)	2.19 (1.87–2.51)
40 to <55 years	4.21 (3.68–4.75)	5.60 (4.90–6.30)
55 to <65 years	6.41 (5.64–7.18)	8.44 (7.46–9.43)
65 to <75 years	11.34 (10.10–12.59)	14.67 (13.11–16.22)
75 to <85 years	15.18 (13.41–16.95)	19.36 (17.18–21.54)
≥85 years	23.99 (20.52–27.46)	29.72 (25.68–33.75)
Males		
0 to <40 years	2.46 (2.10–2.81)	3.29 (2.83–3.76)
40 to <55 years	6.26 (5.51–7.01)	8.25 (7.29–9.20)
55 to <65 years	9.40 (8.36–10.43)	12.24 (10.94–13.53)
65 to <75 years	16.17 (14.56–17.78)	20.55 (18.61–22.50)
75 to <85 years	21.21 (18.93–23.48)	26.51 (23.82–29.19)
≥85 years	32.12 (27.93–36.31)	38.79 (34.16–43.41)

Abbreviations: CI, confidence interval; SARS-CoV-2, severe acute respiratory syndrome coronavirus 2.

### Case Fatality Given Hospital Admission

There were 669 deaths among people admitted to a hospital (alpha: 369 [13.6%]; wild-type: 300 [13.7%]). In fully adjusted analysis, accounting for demographic factors, regional variation, and individual-level comorbidities, alpha was associated with 44% increased hazards of death (aHR: 1.44; 95% CI: 1.11–1.87; *P* = .0057) when compared with wild-type after conditioning on hospital admission (Figure 2). The increased hazard of death for alpha conditional on hospital admission was consistent across all predefined subgroups and sensitivity analyses (Supplementary Figure 2).

Among people admitted to a hospital, 615 of 4910 (12.5%) were admitted to the ICU (alpha: 344; wild-type: 271). Compared with people admitted to a hospital, those admitted to the ICU were of similar age (median age: 59.0 vs 58.0 years), with a higher proportion having 1 comorbidity (1 comorbidity: 31.5% vs 27.4%; ≥2 comorbidities: 19.5% vs 21.9%). Mortality among those admitted to the ICU was high (alpha: 147/344 [42.7%]; wild-type: 99/271 [36.5%]). In fully adjusted analysis, accounting for demographic factors, regional variation, and individual-level comorbidities, the association between alpha and increased mortality was smaller and the CI included the null (aHR: 1.20; 95% CI: .74–1.95; *P* = .45) compared with wild-type cases after conditioning on admission to the ICU (Figure 2).

## DISCUSSION

This study describes the relative severity of the alpha SARS-CoV-2 variant compared with wild-type virus at each stage on the pathway from testing positive to hospital admission and death. The results confirm that alpha causes more severe outcomes, with a 73% increased hazard of death and a 62% increased hazard of hospitalization following a positive test in the community. These findings were consistent across all prespecified sensitivity analyses, including epidemiological week of infection, meaning they cannot be explained by changing eligibility or external phenomena such as hospitals exceeding capacity. These results are in agreement with previous studies that have shown the alpha variant to be associated with higher case fatality in large populations selected based upon positive tests in the community [4–6, 8, 13].

By following people through the pathway of disease, we are able to describe the weakening association between the alpha variant and mortality as the study population is conditioned on more severe disease. When conditioning on hospital admission, alpha was associated with 44% increased hazards of death; when further conditioning on disease severe enough to require admission to the ICU, there was no evidence that alpha was associated with higher case fatality than wild-type virus, although power for this analysis was limited and the CI was consistent with the estimate from the full study population.

Studies among people admitted to the ICU may not provide reliable estimates of relative case fatality.

These findings are in agreement with studies that have assessed relative case fatality of the alpha variant among hospitalized patients [9, 14]. However, there is no contradiction in the results showing increased mortality for alpha in the community, but no evidence of increased mortality for alpha among those admitted to the ICU. Risk factors for death following SARS-CoV-2 infection have been described in detail elsewhere [15], with the predominant risk factors being older age and the presence of comorbidities. Conditioning on hospital admission controls for these risk factors to some extent, as seen by the older age and higher prevalence of comorbidities among hospitalized patients. Further, people admitted to the ICU are a complex study population. In order to be admitted to the ICU the attending clinician must consider the illness to be severe enough to require intensive care, but also that the person has a reasonable chance of survival. Therefore, people with alpha and wild-type virus admitted to the ICU may be predisposed to similar case fatality.

It remains the case that, even if there is no difference in relative mortality among people with alpha and wild-type virus admitted to the ICU, a variant that results in more people being admitted to a hospital and the ICU will have higher case fatality.

In Frampton et al [9], who studied 341 patients hospitalized with SARS-CoV-2, no association between alpha and increased mortality was found. These findings support the above reasoning as their population was selected from acutely admitted and severely ill patients. Further, although no evidence of a difference in mortality risk was found, the group with the alpha variant had higher viral load, were younger, and had fewer comorbidities, which is consistent with more severe disease.

The absolute risk estimates for death and hospitalization following a positive test presented here relate specifically to an unvaccinated population as they are derived from a time when vaccination against SARS-CoV-2 was rare and vaccination prior to infection was an exclusion criterion. These estimates provide important context for assessing the severity of future variants and the impact of the vaccination campaign on the ongoing pandemic. Recent estimates from Public Health England indicate that 2 doses of Pfizer or Oxford-AstraZeneca vaccine against SARS-CoV-2 reduce the risk of hospitalization by more than 90% [16]. From our data on the alpha variant, this would result in a reduction in the number of hospitalizations among females under the age of 40 with no comorbidities from 7 in 1000 to 7 in 10 000. Among males over the age of 85 years with 2 or more comorbidities, the reduction would be from 39 in 100 to 3.9 in 100.

The strengths of our study include the large study population with individual-level data from primary care on coded diagnoses, medications, vaccinations, and physiological parameters. Linking these data to key datasets, such as ONS deaths data, means we have complete outcome determination for our study period. The main limitation of the analysis is that alpha and wild-type viruses are determined by the SGTF proxy, which is less accurate for variant determination than sequencing. However, analysis indicates the sensitivity of SGTF over the study period is over 95%, and previous work has shown that the prevalence of SGTF in the OpenSAFELY study population is representative of England [6].

Spike gene target failure data are available only for people testing positive for SARS-CoV-2 in the community; as a result, people with mild or asymptomatic infections who do not present for testing are not included, which may result in overestimation of the absolute risks of death and hospital admission. In addition, SARS-CoV-2 tests performed in hospitals in England are not tested for SGTF; consequently, people tested first in a hospital (ie, in emergency departments or on admission) are not included despite being likely to have more severe disease than those tested in the community.

Our study shows that the SARS-CoV-2 alpha variant causes more severe disease than wild-type virus following the pathway of illness from testing positive to hospital admission and death.

## Supplementary Data

Supplementary materials are available at *Clinical Infectious Diseases* online. Consisting of data provided by the authors to benefit the reader, the posted materials are not copyedited and are the sole responsibility of the authors, so questions or comments should be addressed to the corresponding author.

ciab754_suppl_Supplementary_Materials

## Notes

***Acknowledgments.*** The authors are grateful for the support received from the TPP Technical Operations team and for generous assistance from the information governance and database teams at NHS England/NHSX.

***Financial support.*** This work was jointly supported by UK Research and Innovation (UKRI), the National Institute for Health Research (NIHR), and Asthma UK–British Lung Foundation (COV0076; MR/V015737/), and the Longitudinal Health and Wellbeing strand of the National Core Studies program. The OpenSAFELY data science platform is funded by the Wellcome Trust. TPP provided technical expertise and infrastructure within their data center pro bono in the context of a national emergency. R. M. E. is funded by Health Data Research UK (HDR UK) (grant number MR/S003975/1), the Medical Research Council (MRC) (grant number MC_PC 19065), and NIHR (grant number NIHR200908).

***Potential conflicts of interest.*** E. W. has received payment from AstraZeneca and Roche for providing training and advice, unrelated to the current work. H. I. M. is funded by the NIHR Health Protection Research Unit (HPRU) in Vaccines and Immunisation and has been an unpaid invited expert (expert advisory group [EAG]) to the Commission on Human Medicines (CHM) COVID-19 Vaccines Safety Surveillance Methodologies Expert Working Group. L. S. reports institutional research grants from UKRI, NIHR, and Wellcome, during the conduct of the study. N. J. D. reports grants/support from BMBF, Fetzer Franklin Fund, Good Thinking Society, and Laura and John Arnold Foundation (these are all grants outside the submitted work that should not present any direct conflicts) and travel support from Kellogg College and Nuffield Department of Primary Care Health Sciences (these are their department and college at Oxford and should not represent any conflict with this work). R. M. reports grants/contracts/support from Wellcome Trust, serving on an advisory board/DSMB for Wellcome Trust, and receiving consulting fees from AMGEN, outside the submitted work. R. M. E. reports grants from UKRI and HDR UK (all standard funding agency grants to the institution) during the conduct of the study and grants from IMI2 (standard funding agency grant to the institution), outside the submitted work. B. G. reports support from Wellcome, MRC/NIHR, and NCS LHW (OpenSAFELY platform grants), during the conduct of the study; B. G. has received research funding from the Laura and John Arnold Foundation, the NHS NIHR, the NIHR School of Primary Care Research, the NIHR Oxford Biomedical Research Centre, the Mohn-Westlake Foundation, NIHR Applied Research Collaboration Oxford and Thames Valley, the Wellcome Trust, the Good Thinking Foundation, Health Data Research UK, the Health Foundation, the World Health Organization, UKRI, Asthma UK, the British Lung Foundation, and the Longitudinal Health and Wellbeing strand of the National Core Studies program, outside the submitted work. B. G. reports personal income from journalism, public speaking, and popular science books on the misuse of science. B. G. reports the following leadership roles: United Kingdom Government, Department for Health and Social Care HealthTech Advisory board chair, leading review for the Secretary of State, as Non-Executive Director of NHS Digital. K. B. reports a fellowship with Wellcome Trust and payment to his institution, during the conduct of the study. A. S. reports being employed by LSHTM on a fellowship sponsored by GSK outside of the submitted work. I. J. D. reports grants to LSHTM from GSK and GSK shares outside of the submitted work. L. T. reports grants or contracts from Wellcome Trust, MRC, and NIHR; support for an MHRA Expert Advisory Group × 4; and being a member of 2 non–industry-funded trial advisory committees (unpaid), all outside of the submitted work. All other authors report no potential conflicts. All authors have submitted the ICMJE Form for Disclosure of Potential Conflicts of Interest. Conflicts that the editors consider relevant to the content of the manuscript have been disclosed.
